# Changes in waist circumference and the prevalence of abdominal obesity during 1994–2008 - cross-sectional and longitudinal results from two surveys: the Tromsø Study

**DOI:** 10.1186/s40608-016-0121-5

**Published:** 2016-09-21

**Authors:** Bjarne K. Jacobsen, Nils Abel Aars

**Affiliations:** Department of Community Medicine, UiT – The Arctic University of Norway, Tromsø, Norway

**Keywords:** Waist circumference, Obesity, Abdominal, Epidemiology

## Abstract

**Background:**

Abdominal obesity increases all-cause mortality and is a risk factor for a number of diseases. There are few population-based studies of the longitudinal changes of abdominal obesity.

**Methods:**

Based on data from the Tromsø Study, we studied gender- and age-specific mean waist circumference and prevalence of abdominal overweight and abdominal obesity in two surveys in 1994–1995 (Tromsø 4, 6812 men and women aged 25 to 84) and 2007–2008 (Tromsø 6, 12,493 men and women aged 30 to 87). Furthermore, we describe the longitudinal changes of waist circumference and abdominal obesity during 13 years in 3144 subjects (aged 25–69 in 1994) who attended both surveys.

**Results:**

Cross-sectional analyses found a higher mean waist circumference in men than women and a direct relationship with age in both men and women in both Tromsø 4 and in Tromsø 6. As the WHO cut-off points for abdominal obesity are gender-specific, however, the prevalence of abdominal obesity was lower in men than in women. In 2007–2008, approximately 37 and 55 % of men and women, respectively, were classified as abdominally obese. Thirteen years before, in 1994–1995, the corresponding figures were 20 and 35 %. Longitudinal analyses of changes during the 13-year period clearly demonstrated that mean waist circumference increased in all examined birth cohorts in both men (mean change 6.1 cm) and women (mean change 8.4 cm), but increased more markedly the younger the subjects were. The prevalence of abdominal obesity in men aged 25–34 increased from 5 % in 1994 to 31 % 13 years later. The prevalence of abdominal obesity more than doubled among both men and women.

**Conclusions:**

The increasing mean waist circumference is of concern. There is a need for further longitudinal studies of the changes in waist circumference.

## Background

Obesity and overweight is often assessed by the body mass index (BMI), but other measures, like waist circumference, may be equally, or even more, informative with regard to the associated health risks. Abdominal obesity, measured as waist circumference, has been found to be a significant predictor of obesity related diseases [1, 2] and all-cause mortality [3–5]. Information about the prevalence of abdominal obesity is therefore of major public health concern.

Some studies indicate that the waist circumference may have increased more than the increase in body mass index can explain [6–9]. According to US data, the mean waist circumference still increases even if mean body mass index may not increase as much anymore [10]. Visscher et al. have recently discussed the possible break in the obesity epidemic and concluded that this at least does not apply to waist circumference [11].

Cross-sectional studies have found waist circumference to increase with age [7, 12–14]. Longitudinal analyses of changes in body mass index have shown that the highest weight increase takes place in the younger age groups [15–22], and similarly, some longitudinal studies [23, 24] have reported that the increase in waist circumference is higher the younger the people were; that there is a birth cohort effect in addition to a secular effect.

We have previously reported an increase in body mass index in all included 5-year birth cohorts in the Tromsø municipality during the 1974–1994 time span [15]. Recently, we extended the analyses to the period 1994–2008 [22]. Data concerning waist circumference were not available, however, before 1994. In the present study, we report the cross-sectional results from 1994 (including 6812 men and women) and 2008 (including 12,493 men and women) as well as changes in waist circumference between 1994 and 2008, including longitudinal changes in a subgroup of 3144 individuals.

## Methods

The Tromsø Study is a large population study, based on the population of Tromsø in the north of Norway. It was conducted for the first time in 1974 (Tromsø 1) and the surveys have been repeated six times [25, 26]. The present analyses are based on the 4^th^ and 6^th^ Tromsø surveys.

The 4^th^ Tromsø Survey (Tromsø 4) took place in 1994–1995 and the entire population of Tromsø aged 25 or more, 37,558 men and women, was invited, and 27,158 (72 %) attended. All participants received a questionnaire with the invitation. The clinical examination included measurements of height and weight. Individuals aged 55–74, as well as samples of 5-10 % in the remaining age groups 25–54 and 75–84 were eligible for a second visit, which included measurements of waist- and hip circumference. A total of 6902 men and women (76 % of the eligible population) attended the second visit.

The 6^th^ Tromsø Survey (Tromsø 6) [26] was conducted in 2007–2008. The 12,984 participants (out of 19,762 invited, 66 %) were invited from 4 different groups: participants from the second visit in Tromsø 4, a 10 % random sample of the age group 30–39, everyone in the age groups 40–42 and 60–87, and a 40 % random sample of people aged 43–49 years The clinical examination included weight, height, waist- and hip circumference. Waist circumference was selected as the measure of abdominal obesity in the analyses. In addition, two other indices, waist-to-height ratio and waist-to-hip ratio, were computed.

Waist circumference was measured across the belly button wearing light clothing and recorded to the nearest cm (in Tromsø 4, to the nearest 0.5 cm) by trained staff using a tape measure with the subject standing and breathing normally [26].

Abdominal overweight was defined as waist circumference 95–102 cm in men and 81–88 cm in women. Abdominal obesity was considered present if waist circumference was > 102 cm in men and > 88 cm in women [5, 27]. It has been found that these cut-offs may be too low for individuals aged 70 and above, and we have therefore, in a separate set of analyses, applied 106 cm in men and 99 cm in women as cut-offs for abdominal obesity in subjects aged ≥ 70 years as suggested by Heim et al. [28].

Information about current pregnancy was obtained by questionnaires and interview. For the present cross-sectional analyses, we selected men and non-pregnant women with valid data about waist circumference who consented to their data being included in research. A total of 6812 men and women were included in the cross-sectional analyses of waist circumference based on the Tromsø 4 survey. The corresponding figure for Tromsø 6 was 12,493 men and women.

The longitudinal analyses of the changes in waist circumference included non-pregnant subjects aged 25–69 in the Tromsø 4 (1994–1995) survey with measurements of waist in both Tromsø 4 (1994–1995) and Tromsø 6 (2007–2008) surveys. Older subjects (aged 70 and above in 1994) were excluded as only 186 out of 1175 older subjects (16 %, all aged 70–74 in 1994) who attended the survey in 1994, also attended the survey 13 years later. The mean age of these 186 subjects was in 2007 84.5 years and we assume that they were particularly healthy. Thus, 3144 subjects were followed with regard to waist circumference.

Fifty-six percent of subjects aged 25–69 who had their waist circumference measured in 1994–1995 (Tromsø 4) also took part in the survey 13 years later (Tromsø 6). These subjects had both been invited to Tromsø 6 (belonged to the invited cohort, had not died or moved out of Tromsø after Tromsø 4) and attended, if invited. The mean waist circumference among the 3144 men and women in Tromsø 4 with a waist circumference measurement in both Tromsø 4 and Tromsø 6 was compared to the waist circumference of the 2493 attenders in Tromsø 4 who lack data from Tromsø 6. In men, there was a tendency towards a lower circumference (0.7 cm age-adjusted) in subjects who attended both surveys (*p* = 0.06 after adjustment for age). In women, a 1.5 cm age-adjusted difference in the same direction was found (*p* < 0.001). When stratified for 10-years age group (as in Table 2), the only group with a statistically significant (*p* < 0.05) difference in body mass index between subjects who attended both Tromsø 4 and Tromsø 6, and those who did not, was women aged 55–64 (2 cm lower waist circumference in women who attended both surveys, *p* < 0.001). Our findings therefore indicate that the longitudinal cohort is quite representative with regard to waist circumference at baseline of the subjects with measurements of waist circumference in Tromsø 4.

The Tromsø Study was approved by the Regional Committee for Research Ethics. All participants gave written consent.

### Statistical analyses

Age is given as age in years per 31.12.1994 for Tromsø 4 and per 31.12.2007 in Tromsø 6.

The age categories used in the cross-sectional analyses were 25–34 (in Tromsø 6: 30–34), 35–44, 45–54, 33–64, 65–74, 75–84 and (in Tromsø 6) 85–89 years old. In the longitudinal analyses, the age groups were (age in Tromsø 4): 25–34, 30–34, 35–44, 45–54, 55–64, 65–69.

The measurement of waist circumference is more difficult to standardize than measuring height and weight [29]. In a separate set of the longitudinal analyses, a z-score analysis was conducted in order to avoid the effect of any possible systematic differences in how the measurements had been performed in the different surveys. Z-scores were computed for men and women separately for those with valid measurements of waist circumference in Tromsø 4 and Tromsø 6. The z-score was computed, separately for each survey, as measured waist circumference minus mean waist circumference, divided by the standard deviation of waist circumference. The z-scores for Tromsø 4 and Tromsø 6 according to age in Tromsø 4 were compared, enabling assessment of the changes in waist circumference in relative rather than absolute terms.

As the age-distribution in Tromsø 4 and Tromsø 6 (Table 1) differs, direct age-adjustment was performed, including the age group 35–74 (few subjects were younger or older). The age-adjusted estimates for mean waist circumference and the prevalence of abdominal overweight and obesity in Tromsø 4, given the age-distribution in the Tromsø 6 (the larger survey) were estimated.

**Table 1:** Waist circumference, prevalence of abdominal overweight^a^ and obesity^b^ in 1994–1995 and 2007–2008. The Tromsø Study

	Tromsø 4 (1994–1995)				Tromsø 6 (2007–2008)			
Men								
Age	n	Mean (SD)^d^	% overweight	% obese	n	Mean (SD)	% overweight	% obese
25/30–34^c^	104	88.1 (8.1)	9.6	6.7	86	95.9 (9.8)	37.2	18.6
35–44	137	91.0 (7.6)	20.4	8.0	1,131	97.5 (10.6)	29.0	29.6
45–54	457	96.2 (8.9)	33.0	23.0	1,068	98.3 (10.5)	31.9	31.0
55–64	1,511	95.2 (9.1)	33.0	19.1	1,733	100.4 (10.4)	29.9	40.9
65–74	1,121	95.7 (9.7)	29.3	23.8	1,314	100.7 (10.4)	30.6	41.0
75–84	34	95.2 (10.7)	32.4	23.5	457	100.8 (10.7)	29.3	42.0
85–89	-	-	-	-	32	99.3 (11.5)	18.8	46.9
Total	3,364	95.1 (9.4)	30.5	20.4	5,821	99.5 (10.6)	30.3	36.7
Total 35–74	3,226	95.3 (9.3)	31.2	20.8	5,246	99.4 (10.6)	30.3	36.5
Total 35–74 adjusted^e^		94.6	29.4	18.7		99.4	30.3	36.5
Women								
25/30–34^c^	117	77.5 (9.5)	12.8	12.0	119	85.7 (11.4)	26.1	36.1
35–44	174	79.0 (9.8)	19.0	14.4	1,381	88.6 (12.1)	27.0	44.4
45–54	198	80.7 (8.6)	23.7	19.2	1,244	89.7 (12.2)	26.8	49.4
55–64	1,559	84.5 (10.7)	32.8	30.3	1,854	92.2 (12.1)	23.6	59.8
65–74	1,354	87.6 (11.3)	26.3	45.2	1,338	92.1 (11.9)	22.9	60.3
75–84	46	92.0 (14.3)	19.6	56.5	671	93.0 (11.8)	21.6	63.8
85–89	-	-	-	-	65	92.3 (11.5)	16.9	66.2
Total	3,448	85.1 (11.1)	28.2	34.5	6,672	90.9 (12.2)	24.5	54.8
Total 35–74	3,285	85.3 (11.0)	26.8	35.0	5,817	90.8 (12.2)	24.9	54.0
Total 35–74 adjusted^e^		83.1	26.1	27.6		90.8	24.9	54.0

^a^Waist circumference 95–102 cm in men and 81–88 cm in women ^b^Waist circumference > 102 cm in men and > 88 cm in women

^c^Age group 25–34 in Tromsø 4 (1994–1995), 30–34 in Tromsø 6 (2007–2008) ^d^centimeter (standard deviation)

^e^Mean waist circumference and prevalence of overweight and obesity in the age group 35–74 adjusted to the age distribution in Tromsø 6

In order to investigate in the longitudinal analyses whether the change in waist circumference was over and above that expected based on increased body mass index and age from Tromsø 4 to Tromsø 6, regression analysis was conducted. We assumed that the relationship between waist circumference (the dependent variable) and BMI and age (the two independent variables) based on the Tromsø 4 data set also was valid for the same subjects 13 years later (in Tromsø 6) and computed the expected waist circumference in Tromsø 6, which was compared with the observed waist circumference.

The results from the longitudinal analysis are presented according to age in the Tromsø 4 (in 1994–1995) survey, while the results from the cross-sectional analyses are presented according to age groups in each survey.

The analyses are based on the much larger Tromsø Study database, and each project based on it has to be authorized and data cannot be shared.

All analyses were conducted using SAS version 9.4. The statistical analyses included simple descriptive analyses, Chi square test, logistic regression, independent sample *t*-test and linear regression. A *p*-value of < 0.05 was considered statistically significant.

## Results

The two other possible measures of abdominal obesity (waist-to-height ratio and waist-to-hip ratio) were statistically highly significantly correlated to waist circumference; *r* > 0.92 for waist-to-height ratio and *r* > 0.7 for waist-to-hip ratio in both men and women in Tromsø 4 and Tromsø 6.

Table 1 gives the results from the cross-sectional studies Tromsø 4 and Tromsø 6.

The mean waist circumference was consistently higher in men than in women, and the mean circumference and the prevalence of abdominal obesity increase with age in both men and women and in both surveys (*p* < 0.001). We noticed, however, a tendency toward a lower mean waist circumference in the oldest subjects, aged 85–89, in Tromsø 6. There were in both men and women statistically significant relationships between age group and the distribution of the respondents according to normal waist circumference, abdominal overweight and abdominal obesity; the tendency to being classified as overweight/obesity was positively associated with increasing age.

In Tromsø 6, the majority of the population, even in the younger age groups, was classified as abdominally overweight or obese (Table 1). The mean waist circumference and the proportion classified as obese increased in both genders from Tromsø 4 to Tromsø 6 in every examined age-group. The age-adjusted (age 35–74) proportion of the population classified as abdominal obese increased in men from 18.7 % in Tromsø 4 to 36.5 % in Tromsø 6. The corresponding figures for women were 27.6 to 54.0 % (Table 1).

When we applied higher cut-offs for subjects aged 70 and above (106 cm in men and 99 cm in women), the prevalence of abdominal obesity in men was 18.6 % in the Tromsø 4 survey and 34.4 % in the Tromsø 6 survey. The corresponding figures for women were 28.4 % (Tromsø 4) and 48.0 % (Tromsø 6).

Table 2 provides the longitudinal results for men and women aged 25–69 (i.e., born 1925–1969) in the Tromsø 4 survey who took part in both the Tromsø 4 and Tromsø 6 survey. The waist circumference increased in all age groups and a statistically significant (*p* < 0.001) inverse relationship was found between age in Tromsø 4 and the change in waist circumference between Tromsø 4 and Tromsø 6. Analyses applying z-scores of the waist circumference rather than the actual waist circumference confirmed these findings of a general inverse relationship between age and change in waist circumference during the 1994 to 2008 period (results not shown in the tables).

**Table 2:** Longitudinal changes in waist circumference, prevalence of abdominal obesity^a^ between 1994–1995 and 2007–2008 in 3,144 subjects. The Tromsø Study

		Tromsø 4 (1994–1995)		Tromsø 6 (2007–2008)		Changes between Tromsø 4 and Tromsø 6	
Age in 1994 (birth year)	n	Mean (SD)^b^	% obese	Mean (SD)	% obese	Mean (95 % CI)^c^	Absolute change in % obese (relative to 1994, %)
Men							
25–34 (1960–69)	62	88.4 (7.8)	4.8	98.2 (10.0)	30.7	9.8 (7.9, 11.6)	25.8 (+538 %)
35–44 (1950–59)	89	90.9 (7.4)	7.9	98.2 (9.1)	33.7	7.3 (5.8, 8.8)	25.8 (+327 %)
45–54 (1940–49)	326	96.1 (8.5)	21.8	102.3 (10.5)	46.6	6.2 (5.5, 7.0)	24.8 (+114 %)
55–64 (1930–39)	850	94.8 (8.2)	18.0	100.6 (10.2)	40.4	5.8 (5.3, 6.3)	22.4 (+124 %)
65–69 (1925–29)	182	95.1 (7.8)	17.6	100.8 (11.0)	43.4	5.7 (4.7, 6.8)	25.8 (+147 %)
Total	1,509	94.6 (8.3)	17.6	100.8 (10.4)	41.3	6.1 (5.8, 6.5)	23.7 (+135 %)
Women							
25–34 (1960–69)	76	76.9 (9.1)	10.5	86.3 (10.1)	36.8	9.4 (7.4, 11.4)	26.3 (+250 %)
35–44 (1950–59)	134	79.2 (9.9)	14.9	91.3 (12.3)	57.5	12.1 (10.6, 13.6)	42.5 (+285 %)
45–54 (1940–49)	158	80.7 (8.6)	17.7	90.2 (10.0)	58.2	9.6 (8.3, 10.8)	40.5 (+229 %)
55–64 (1930–39)	973	83.8 (9.8)	26.6	91.9 (11.6)	61.4	8.1 (7.5, 8.6)	34.7 (+130 %)
65–69 (1925–29)	294	86.5 (9.7)	42.9	93.3 (12.0)	64.0	6.8 (5.8, 7.8)	21.1 (+49 %)
Total	1,635	83.3 (9.9)	27.0	91.7 (11.6)	60.1	8.4 (8.0, 8.8)	33.1 (+123 %)

^a^Waist circumference > 102 cm in men and > 88 cm in women

^b^centimeter (standard deviation)

^c^Mean change in centimetre (95 % confidence interval)

The longitudinal analyses further demonstrated that the proportion of the population that was classified as abdominally obese more than doubled in both men and women during 1994–2008, and there was a 6–7 % mean annual increase in the prevalence of abdominal obesity. In men, there were no relationships between age in Tromsø 4 and the number of percent points the prevalence of abdominal obesity increased (e.g. 25.8 %-points in men aged 25–34 and in men aged 65–69 in Tromsø 4). In women, a linear inverse relationship was noted (*p* = 0.006) as the prevalence of abdominal obesity increased by 36.6 %-points in women aged 25–34 and 21.1 % in the oldest women (aged 65–69). However, in relative terms, the prevalence increased much more in the younger age groups (e.g., more than 500 % from 4.8 to 30.7 % in men aged 25–34 in 1994) than in the older age groups (147 % from 17.6 to 43.4 % in men aged 65–69). The lowest relative increase (49 %, from 42.9 to 64.0 %) was seen in the oldest age group in women, but they also had the highest prevalence of abdominal obesity in Tromsø 4.

The correlation coefficient between waist circumference in Tromsø 4 and Tromsø 6 was 0.7 for both men and women. In men, we found that 88 % of the initially obese (waist circumference > 102 cm) (in Tromsø 4) were also obese 13 years later and only 1 % had normal waist circumference (<95 cm). In accordance with the increased mean waist circumference in the population, a significant proportion (16 %) of men initially classified as having normal waist circumference (<95 cm) were obese in Tromsø 6. In women, 91 % of the initially obese (waist circumference > 88 cm) (in Tromsø 4) were also obese 13 years later and 3 % had normal waist circumference (<81 cm). Thirty-two % of women initially classified as having normal waist circumference were obese in Tromsø 6.

In both men and women, a significantly larger waist circumference was observed in Tromsø 6 than expected based on the increased body mass index and advancing age. For men, the difference between the observed and expected waist circumference was 2.1 cm (95 % CI: 1.8–2.4 cm) and in women 5.1 cm (4.8–5.4 cm). Our data clearly indicate that most of the statistical relationship between age and change in waist circumference may be explained in statistical terms by the relationship between age and changes in body mass index.

## Discussion

We find that the mean waist circumference in the Tromsø population has increased from 1994 to 2008 and that longitudinal analyses demonstrate the increase is inversely related to the age; the younger (aged 25–34) increase their waist circumference more than older people (aged 65–69) do. Thus, our results from a relatively large population-based study confirm some earlier longitudinal studies [23, 24]. The relationships between age and the longitudinal changes for waist circumference are similar to those found for the changes in body mass index (e.g. [15, 17, 22]) and may, according to our results, to a large extent be explained by them.

As only data from two points in time, 1994–1995 and 2007–2008, are included in the analyses, we were not able to examine whether there the increase in waist circumference was less marked in the last part of these 13 years, which was the case for body mass index [22]. There is, however, little evidence from other studies, that such a levering off has taken place for the mean waist circumference and central obesity [7, 10, 11, 30, 31]. There is a need for more longitudinal studies with measurements from several points in time.

Some cross-sectional results regarding the waist circumference in the Tromsø 6 survey (2007–2008) have been published [26], but not in any detail. The prevalence of abdominal obesity in Tromsø 6 (2007–2008) was somewhat lower than for non-Hispanic white subjects in the US [32]. The US data were based on the NHANES, and the waist circumference was measured just above the iliac crest. In men, the prevalence of abdominal obesity was somewhat higher than in another Norwegian population study, the HUNT study [14], which used very similar screening methods and was conducted at the same time as Tromsø 4 and Tromsø 6 (HUNT 2 in 1995–1997 and HUNT 3 in 2006–2008).

In this Tromsø population, the prevalence of obesity as assessed by general obesity (BMI) [22] and abdominal obesity (waist circumference) (the present study) differs considerably, the latter being higher. This is in accordance with previous studies [10, 14, 33] and may indicate that the WHO criteria for general and abdominal obesity need to be harmonized. In the analyses, we chose to concentrate on waist circumference rather than e.g., waist-to-hip ratio or waist-to-height ratio [34] as the measure of abdominal obesity. The primary reason was that both these measures of anthropometry are strongly correlated to waist circumference.

We also note that there is a strong tracking for abdominal obesity, as we have previously demonstrated for general obesity [35] and that the increase in waist circumference during 1994–2008 was for both men and women significantly larger than can be expected from the increases in body mass index and age. This is in accordance with findings from some previous studies [8, 9].

### Strengths and limitations

Among the strengths of the present study is the relatively high attendance rate in the Tromsø Study (76 % in the 1994–1995 survey and 66 % in the survey conducted in 2007–2008). It is also a significant strength that all the data concerning waist circumference were based on measurements using standardized procedures. However, waist circumference is prone to measurement error [29], and to avoid the effect of any possible systematic differences in how the circumference were measured at the surveys, z-scores were computed. When comparing the longitudinal results based on the actual measured waist circumference and the z-score analyses, the conclusions regarding the longitudinal changes were unchanged. This further strengthens the results from the longitudinal analyses of the waist circumference.

There are also limitations, however. It is well known that attenders to a health survey tend to differ from non-attenders as the latter group generally has more health problems, higher mortality and were of lower socioeconomic status. This has been found both in the Tromsø Study [25, 36] and in similar studies in Norway [37, 38].

Furthermore, subjects who attended both the Tromsø 4 and Tromsø 6 survey had in Tromsø 4 1.1 cm (1.2 %) lower waist circumference than subjects with information from Tromsø 4, but not from Tromsø 6. These were subjects who were not invited to the survey, chose not attend, had moved out of Tromsø or had died. This relatively minor difference in waist circumference, although statistically significant, is of particular importance for longitudinal analyses presented in this study. We consider that it is unlikely that any major bias has been introduced, but we cannot exclude that selective attrition has had an impact on our findings, particularly in the older age groups. If men and women with high waist circumference in Tromsø 4 died or chose not to attend 13 years later, a relatively low increase in the waist circumference from Tromsø 4 to Tromsø 6 will be the result in the subjects available for the presented analyses. However, even if subjects aged 65–69 in Tromsø 4, i.e., men and women aged 78–82 in Tromsø 6, were excluded from the analyses, the inverse relationship between age in 1994 and change in waist circumference the following 13 years is convincing (Table 2).

The waist circumference may be measured in different ways (like at the level of belly button, the top of the iliac crest, or the minimal waist circumference [5]). Assessment of waist circumference is, as noted above, more difficult to standardize than e.g., measurement of height and weight [5, 29]. This has without doubt resulted in misclassification and it hampers the comparison with other studies. A further limitation in our study is the low number of subjects in some age groups, particularly in Tromsø 4.

## Conclusions

High waist circumference has been linked to a number of chronic diseases, and the relationships has been considered both strong and convincing by the World Health Organization [5], and an increase in waist circumference has detrimental metabolic consequences [39]. The relationships between waist circumference and mortality may be attenuated in older subjects, though [3, 40]. Our findings from both cross-sectional and longitudinal analyses of an increased mean waist circumference, and particularly that the increase is inversely associated with age, are therefore of concern. In 2008, 37 % of the men and nearly 55 % of the women in the Tromsø population were considered to have abdominal obesity. Thirteen years before, the corresponding figures were 20 and 35 %. There is a need for further longitudinal studies of the changes in waist circumference and the predictors for it.
